# Mapping migratory flyways in Asia using dynamic Brownian bridge movement models

**DOI:** 10.1186/s40462-015-0029-6

**Published:** 2015-02-02

**Authors:** Eric C Palm, Scott H Newman, Diann J Prosser, Xiangming Xiao, Luo Ze, Nyambayar Batbayar, Sivananinthaperumal Balachandran, John Y Takekawa

**Affiliations:** U.S. Geological Survey, Patuxent Wildlife Research Center, Beltsville, MD 20705 USA; Food and Agriculture Organization of the United Nations, Emergency Center for Transboundary Animal Disease, Hanoi, Vietnam; Department of Botany and Microbiology, Center for Spatial Analysis, University of Oklahoma, Norman, OK 73019 USA; Institute of Biodiversity Science, Fudan University, Shanghai, 200433 China; Computer Network Information Center (CNIC), Chinese Academy of Sciences, Beijing, 100080 China; Wildlife Science and Conservation Center, Ulaanbaatar, 210351 Mongolia; Bombay Natural History Society, Hornbill House, Mumbai, 400 001 India; U.S. Geological Survey, Western Ecological Research Center, San Francisco Bay Estuary Field Station, Vallejo, CA 94592 USA; National Audubon Society, Science Division, 220 Montgomery Street, San Francisco, CA 94104 USA

**Keywords:** Dynamic Brownian bridge movement model, Flyways, Waterfowl, Migration, Stopover sites, Space use, Habitat conservation

## Abstract

**Background:**

Identifying movement routes and stopover sites is necessary for developing effective management and conservation strategies for migratory animals. In the case of migratory birds, a collection of migration routes, known as a flyway, is often hundreds to thousands of kilometers long and can extend across political boundaries. Flyways encompass the entire geographic range between the breeding and non-breeding areas of a population, species, or a group of species, and they provide spatial frameworks for management and conservation across international borders. Existing flyway maps are largely qualitative accounts based on band returns and survey data rather than observed movement routes. In this study, we use satellite and GPS telemetry data and dynamic Brownian bridge movement models to build upon existing maps and describe waterfowl space use probabilistically in the Central Asian and East Asian-Australasian Flyways.

**Results:**

Our approach provided new information on migratory routes that was not easily attainable with existing methods to describe flyways. Utilization distributions from dynamic Brownian bridge movement models identified key staging and stopover sites, migration corridors and general flyway outlines in the Central Asian and East Asian-Australasian Flyways. A map of space use from ruddy shelducks depicted two separate movement corridors within the Central Asian Flyway, likely representing two distinct populations that show relatively strong connectivity between breeding and wintering areas. Bar-headed geese marked at seven locations in the Central Asian Flyway showed heaviest use at several stopover sites in the same general region of high-elevation lakes along the eastern Qinghai-Tibetan Plateau. Our analysis of data from multiple Anatidae species marked at sites throughout Asia highlighted major movement corridors across species and confirmed that the Central Asian and East Asian-Australasian Flyways were spatially distinct.

**Conclusions:**

The dynamic Brownian bridge movement model improves our understanding of flyways by estimating relative use of regions in the flyway while providing detailed, quantitative information on migration timing and population connectivity including uncertainty between locations. This model effectively quantifies the relative importance of different migration corridors and stopover sites and may help prioritize specific areas in flyways for conservation of waterbird populations.

**Electronic supplementary material:**

The online version of this article (doi:10.1186/s40462-015-0029-6) contains supplementary material, which is available to authorized users.

## Background

For migratory animals, identifying movement routes and stopover sites is necessary for effective population management and habitat conservation [1,2]. Animals experience a variety of challenges during migration, including adverse weather, unpredictable food availability and geographic barriers such as mountain ranges, deserts and oceans. Some migratory birds spend over half of their annual cycle traveling between breeding and wintering areas, and challenges during these periods contribute to a substantial portion of annual mortality in many species [3-6]. However, there are disproportionately few studies analyzing space use during migration (e.g., [7,8]) relative to those that quantify space use during breeding and wintering periods (e.g., [9,10]).

A collection of avian migration routes, known as a flyway, is often hundreds to thousands of kilometers long and can extend across international borders. A flyway encompasses the entire geographic range between breeding and non-breeding areas of populations, single species or across multiple species, and provides a spatial framework for management and conservation across political boundaries [11]. Waterfowl migration routes are perhaps the best described flyways due to a long history of research and management. The concept of multi-species waterfowl flyways began in North America in the 1930s but has since spread to all major global flyways [12]. In 1935, Lincoln [13] first defined and mapped North American waterfowl flyways based entirely on band return data. These biological flyways formed the basis of administrative flyways in the United States, which were designed to manage populations and set hunting regulations [14]. Through the years, waterfowl flyway management programs in North America have become a unique example of long-term collaboration between wildlife research and management. Outside of the United States and Canada, organized flyway-level efforts to conserve waterbirds began in the 1960s in Eurasia and northern Africa, and the first waterbird flyway maps of western Eurasia were published in 1967 [15,11].

**Figure 1:**
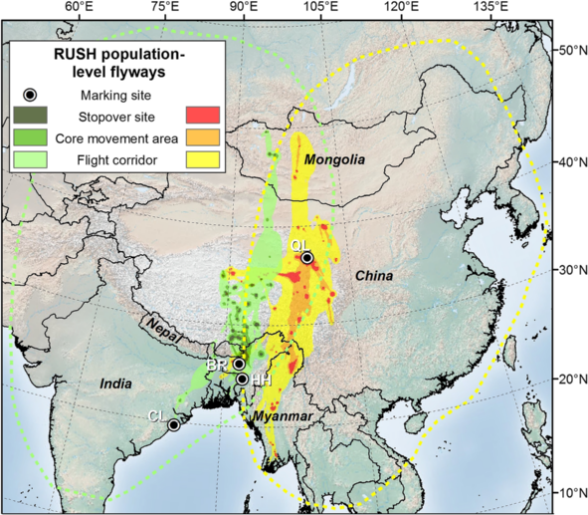
**Estimated migration routes and relative use of ruddy shelducks (RUSH) by population in the CAF.** Data groupings based on geographic proximity of marking sites. From darkest to lightest, colors represent 50%, 75% and 99% cumulative probability contours. Marking sites include Qinghai Lake, China (QL), Brahmaputra River, India (BR), Hakaluki Haor, Bangladesh (HH) and Chilika Lake, India (CL). The western (green) route shows relative use for RUSH marked in northeast India and Bangladesh. The eastern (yellow-red) route shows relative use for RUSH marked at Qinghai Lake, China. Dotted lines represent the RUSH population-level range outlines depicted in Miyabayashi and Mundkur [16].

**Figure 2:**
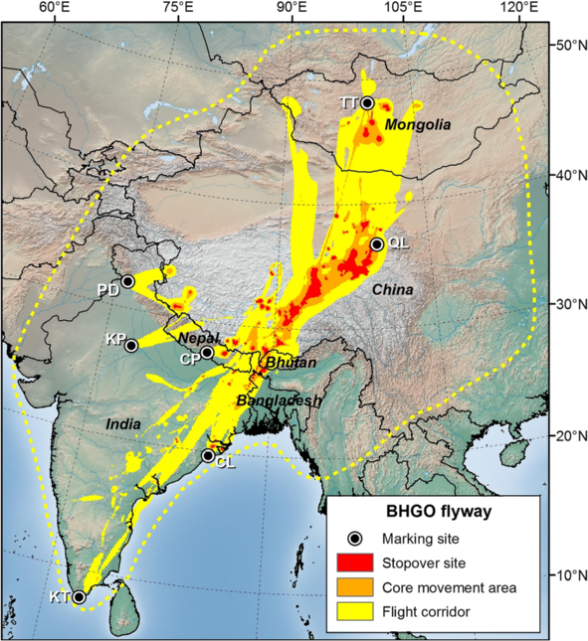
**Estimated migration route and relative use of bar-headed geese (BHGO) in the CAF.** From darkest to lightest, colors represent 50%, 75% and 99% cumulative probability contours. Marking sites include Terkiin Tsagaan Lake, Mongolia (TT), Qinghai Lake, China (QL), Chitwan National Park, Nepal (CP), Pong Dam, India (PD), Keoladeo National Park, India (KP), Chilika Lake, India (CL) and Koonthankulam, India (KT). The dotted line represents the BHGO range outlines depicted in Miyabayashi and Mundkur [16].

**Figure 3:**
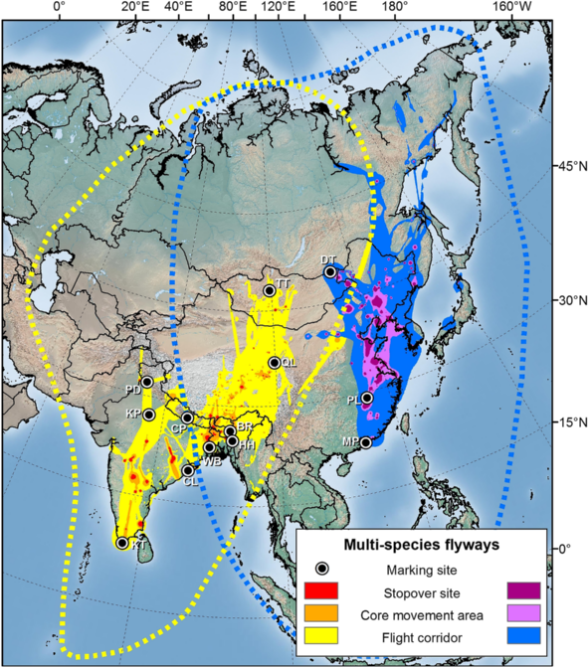
**Estimated migration routes of Anatidae in the CAF and EAAF.** Relative use for CAF displayed in yellow-red and relative use for EAAF displayed in blue-purple. From darkest to lightest, colors represent 50%, 75% and 99% cumulative probability contours. CAF marking sites include Terkiin Tsagaan Lake, Mongolia (TT), Qinghai Lake, China (QL), Chitwan National Park, Nepal (CP), Pong Dam, India (PD), Keoladeo National Park, India (KP), Brahmaputra River, India (BR), Hakaluki Haor, Bangladesh (HH), West Bengal, India (WB), Chilika Lake, India (CL) and Koonthankulam, India (KT). EAAF marking sites include Mai Po, China (MP), Poyang Lake, China (PL) and Delger Tsagaan Lake, Mongolia (DT). Dotted lines represent CAF and EEAF flyway outlines depicted in Miyabayashi and Mundkur [16].

Organized research and flyway-level conservation initiatives in the Asia-Pacific region began much later, and even today, these flyways are only broadly defined and poorly understood [11]. Miyabayashi and Mundkur [16] roughly mapped Anatidae flyways at the species level in the East Asian-Australasian Flyway (EAAF) in 1999 (Figures 1, 2, and 3), but similar information is unavailable for many Anatidae species in the Central Asian Flyway (CAF). The EAAF supports more waterbird species than any other flyway in the world, but >45% of the global human population lives within its boundaries [17], and numerous threats to waterbirds exist including habitat loss from agricultural activities and coastal development, pollution, and hunting [18]. As a result, the EAAF has the highest number of globally threatened waterbird species of any major flyway [17]. In an effort to promote conservation of waterbirds and their habitats in the CAF and EAAF, a flyway-wide coalition of governments and non-government organizations known as the Asia-Pacific Migratory Waterbird Conservation Strategy was established in 1996 [19].

Spatial representations of waterfowl flyways have improved markedly since Lincoln’s first North American maps, but research has yet to incorporate data from satellite telemetry studies to quantitatively describe relative use within flyways. Even the most comprehensive spatial representations of waterfowl flyways are largely qualitative accounts, relying on data from a variety of sources which fail to fully capture connectivity, individual movement routes and relative use within flyways. For example, Bellrose [20,21] highlighted important migration corridors within North American waterfowl flyways by estimating the direction of passage and relative magnitude of birds migrating between different areas within flyways. Bellrose’s maps were based primarily on band return data, which provide little information beyond a start and endpoint, and are inherently biased towards areas with high human population density and hunter activity. Other existing waterfowl flyway maps, such as those in Scott and Rose [22] and Miyabashi and Mundkur [16], are broadly outlined geographic boundaries that encompass a collection of data from a variety of sources, including population monitoring survey data, band return locations, re-sightings of color-marked individuals, and anecdotal accounts.

Beginning in 2006, the United Nations Food and Agriculture Organization (FAO) and the U.S. Geological Survey (USGS) developed a research partnership to assess waterfowl movements throughout Asia and apply models to evaluate their potential to transmit and spread disease. By the end of 2013, the USGS-FAO marking projects had deployed more than 550 satellite transmitters on 26 waterfowl species across 12 countries in Africa and Asia, with a majority in the CAF and EAAF [23].

Satellite and GPS (Global Positioning System) data from the FAO-USGS projects and other avian marking studies provide detailed route information that can improve our knowledge of flyway systems, including data on migration timing, connectivity between breeding and non-breeding areas, migration speed, stopover sites and route fidelity [24]. Researchers have used several methods – most notably, kernel density estimators – to estimate animal space use (i.e., home ranges) from GPS and satellite telemetry data, primarily in breeding and wintering areas. These methods estimate a utilization distribution (UD), which is a probability density representing an animal's relative frequency of occurrence in space and time [25,26]. However, traditional processes for estimating UDs do not account for temporal structure of observations and therefore perform very poorly for actively migrating animals [27].

For migratory species, the Brownian bridge movement model (BBMM) improved upon other methods by estimating a UD based on the animal’s movement path, highlighting both movement corridors and stopover sites. This method calculates the probability of an animal’s use in between telemetry locations by incorporating the distance and elapsed time between successive locations, the location error, and the Brownian motion variance, which estimates an animal’s mobility based on its speed and direction of movement [27]. Sawyer et al. [28] used the BBMM method to estimate population-level migration routes of mule deer. Building on the BBMM, the dynamic BBMM (dBBMM) treats migration movements probabilistically and accounts for temporal autocorrelation in location data. However, instead of assigning a constant Brownian motion variance to all locations in a particular dataset, the dBBMM allows the parameter to vary along the movement path in response to changes in behavior (movement speed) during migration [29]. Specifically in birds, a dynamic variance parameter helps to more accurately distinguish between route segments that function as stopover sites and local movements versus those used primarily as flight corridors.

The objective of this paper is to examine how analyses of satellite and GPS movement data can improve our understanding of migratory bird flyways. We use a new approach to map flyways quantitatively, compare our results to existing range maps and flyway outlines, and present new information on migratory pathways provided by our movement models. Through the FAO-USGS marking program, we use dBBMMs and location data from 141 marked waterfowl to depict examples of probabilistic flyways at the population, species and multi-species levels in the CAF and EAAF. Our examples provide relative space use of bar-headed geese (*Anser indicus*), ruddy shelducks (*Tadorna ferruginea*), and nine other waterfowl species during their semiannual migrations.

## Results and discussion

Our example maps show how the dBBMM can be applied to bird telemetry data to map migration routes and estimate relative use within flyways. Specifically, our results (1) provide evidence suggesting strong connectivity in two separate ruddy shelduck populations, (2) highlight heavily used stopover sites, areas of spatial overlap, and variable migration speeds in bar-headed goose routes, and (3) identify important stopover regions used by multiple species throughout parts of Asia.

### Population-level flyway

Population-level UDs of ruddy shelducks (*n* = 31) showed relative use of two separate migration corridors within the CAF, likely representing two distinct populations that exhibit relatively strong connectivity between breeding and wintering areas (Figure 1; [24]). While all ruddy shelducks migrated along a north-northeast to south-southwest trajectory that bisected the Himalaya Cordillera, birds marked in Bangladesh and northeast India traveled through a corridor several hundred kilometers to the west of those marked at Qinghai Lake. The core use area for birds in the western population included several stopover sites in close proximity to and on both flanks of the Himalayan crest. Ruddy shelducks in the eastern population spent much of the migration period at stopover sites in the vicinity of Qinghai Lake and Madoi County, Tibet. North of the Qinghai-Tibetan Plateau, birds from both populations traveled largely without stops across the Gobi Desert en route to and from breeding grounds in Mongolia. Similarly, birds in the eastern population migrated with few stops from Madoi County south to wintering areas in Myanmar. Although our population-level routes for ruddy shelducks cover a smaller spatial extent than the range outlines found in Miyabayashi and Mundkur [16], our results corroborate their depiction of two separate populations (eastern and western) in the CAF based on census data from the late 1980s and 1990s (Figure 1).

### Single species flyway

In contrast to ruddy shelducks, there was a high degree of spatial overlap among bar-headed goose populations. Our estimated UD aggregating data from 47 bar-headed geese marked at seven sites in the CAF clearly delineates key stopover sites within a heavily traveled migration corridor (Figure 2). Remote, high-elevation lakes along the eastern Qinghai-Tibetan Plateau received the most use and were visited by geese from four of the seven marking sites. The core area of use extended southwest from the Qinghai Lake region towards Lhasa, Tibet and southward to the northernmost extent of Bangladesh. Duration and distance of migration varied by marking site, as described in more detail in Takekawa et al. [30]. Notably, geese marked in the northern and southern limits (Terkhiin Tsagaan Lake, Mongolia, Chilika Lake, India and Koonthankulam, India) stopped more frequently and for longer durations while traveling through the core use area than they did when migrating through these margins. dBBMM UDs also emphasized the individual variation (or lack thereof) in migration paths used by these same birds throughout different parts of their journeys. While flying at the edge of their range, individual geese generally followed separate routes, but they funneled together into a relatively narrow corridor while traveling through the eastern Qinghai-Tibetan Plateau. Birds marked in Nepal and interior northern India bred in the southern Qinghai-Tibetan Plateau, migrating a relatively short distance without major stops. Fifty percent of bar-headed geese marked at Qinghai Lake, China underwent molt migration to an area separate from their breeding area, usually to the southeast. Our comparison of our map to the bar-headed goose range outlined from sightings in Miyabayashi and Mundkur [16] suggests that future marking efforts to the east and west could help provide a more complete picture of the overall flyway (Figure 2).

### Multi-species flyways

Our example of a multi-species flyway map depicting Anatidae migrations in the CAF (*n* = 112) and EAAF (*n* = 29) suggests that the two flyways are spatially distinct and that many stopover areas are used by more than one species (Figure 3). Within the broad outlines depicted in Miyabayashi and Mundkur [16], our results serve as a first attempt to define flight corridors and stopover regions used by multiple species on which future studies can build. Only one bird, a northern shoveler (*Anas clypeata*) marked in Bangladesh that traveled ~6,000 km to eastern Siberia, crossed over from the CAF to the EAAF. Because the majority (66%) of birds in the EAAF (versus 23% in the CAF) analysis were marked with Argos satellite transmitters with larger temporal gaps between successive locations, model outputs in the EAAF showed a higher degree of uncertainty relative to CAF UDs. As a result of this uncertainty, many flight corridors depicted in this map encompass a larger geographic area than if they were derived solely from GPS data. While this uncertainty may have decreased our ability to pinpoint localized stopover sites in the EAAF, our results nonetheless provide valuable information on relative use and connectivity.

### Improving understanding of migratory flyways

Using movement data and dBBMM UDs improves our understanding of migratory flyways by helping to fill many of the gaps in current flyway knowledge. Rather than drawing upon discrete data collection events such as population surveys, band returns and sightings, our flyway examples are based on individual movement paths and provide probabilistic estimates of space use by groups of migrating birds. On their own, satellite telemetry data offer improvements to traditional data types by providing information on timing, individual connectivity, and stopover sites. However, even among studies using satellite telemetry and GPS data, line segments connecting successive locations and/or minimum convex polygons remain the most common method to describe movements during periods of migration (e.g., [8,31,32]). Our probabilistic flyways have advantages over these approaches because they differentiate between areas used as stopover sites, areas that function as flight corridors, and areas that receive little or no use at all. By aggregating multiple UD outputs to create a flyway-wide map, we gain insight into patterns of relative use, timing and connectivity beyond the individual level.

### Considerations for applying the dBBMM approach

The high costs of transmitters, deployment, and data access associated with satellite tracking studies make it difficult to obtain large sample sizes [24,33]. A sensitivity analysis of our data confirmed that our sample sizes are insufficient to be considered representative of entire real-world flyways. In the five separate datasets (2 population-level, 1 species level and 2 multi-species level) we used to create flyway maps, the average percent volume of intersection (%VI) between subsamples of multi-individual UDs and the corresponding overall flyway UD did not reach an asymptote but steadily increased until we included the entire sample in the analysis (see Additional file 1: Figure S1). The curve depicting %VI for ruddy shelducks in India and Bangladesh, where we only marked 8 individuals, had the steepest slope.

We recognize that small sample sizes are a drawback inherent in all satellite tracking studies and that relying on movement data from relatively few birds limits our ability to make strong inferences at the population level and beyond. However, the value in our approach lies in the new information it provides on migratory flyways, and we suggest that studies using dBBMM analyses help strengthen conclusions by supporting results with additional data such as surveys, stable isotopes, band returns, sightings, and genetics data. Alternatively, researchers may be able to overcome small sample sizes by directing marking efforts towards answering more focused questions at the population or species level, rather than deploying transmitters on multiple species across a large spatial extent [33].

### Implications for conservation and management

dBBMM UDs can help inform conservation prioritization at a variety of spatial scales and demographic units, ranging from large-scale, multi-species flyways to more localized, detailed population-level routes. If the goal is to manage for overall waterfowl numbers and species diversity within a flyway, multi-species flyway maps help highlight the most cost-effective conservation options. dBBMM UDs can also identify the relative importance of different molting areas where large numbers of waterfowl aggregate during the post-breeding period and are thereby vulnerable to habitat degradation or anthropogenic disturbance [34]. On the other hand, single-species and population-level UDs help identify important habitats used by a particular species or demographically distinct populations, which could be useful for managing species or populations of special concern.

The multi-species flyway map of the CAF and EAAF highlights the eastern Qinghai-Tibetan Plateau and the Yellow Sea as primary migratory corridors containing important staging and stopover sites (Figure 3). Habitat alteration during recent decades in both of these areas is affecting waterfowl populations. In the Qinghai-Tibetan Plateau, agricultural development and changes to temperature and precipitation regimes may affect reproductive chronology and wintering distribution of waterfowl species [30]. In the Yellow Sea, rapid conversion of intertidal wetland habitat for land reclamation projects coupled with marked waterbird population declines throughout the region have led scientists to identify the area as a high conservation priority [18].

Output UDs from dBBMM analyses could be used to inform conservation in this area by incorporating a variety of spatial analyses involving waterfowl space use, including relationships with habitat types, climate conditions, and disease risk. For example, Takekawa et al. [7] used UDs from BBMM analyses to examine the spatial relationship between migration corridors of Anatidae and outbreaks of highly pathogenic avian influenza in the EAAF, while Byrne et al. [35] used dBBMM UDs to characterize habitat selection patterns in coyotes (*Canis latrans*), white-tailed deer (*Odocoileus virginianus*) and Rio Grande wild turkey (*Meleagris gallopavo intermedia*).

## Conclusions

Empirical estimates of migratory flyways based on satellite tracking data help build upon largely qualitative accounts that have formed the basis of traditional flyway maps. Specifically, the dBBMM improves our understanding of flyways by estimating relative use throughout the flyway, providing detailed, quantitative information on migration timing and population connectivity, and accounting for uncertainty between observed locations. This new approach can be a valuable conservation tool because it goes beyond delineating spatial boundaries of migration routes and provides a more quantitative way to identify important movement corridors, staging and stopover sites, and demographically distinct populations. Conservation planners can use UD layers from dBBMMs in conjunction with other types of supporting data to help inform management decisions and incorporate these layers into various spatial analyses. While our approach offers many improvements over traditional methods for describing flyways, it will not replace these existing flyway data. Instead, dBBMM outputs fill many of the gaps in current flyway knowledge and help prioritize areas for future marking studies, surveys, and conservation efforts.

## Methods

### Capture and Marking

We marked birds at 10 sites in the CAF and three sites in the EAAF (Table 1; Figures 1, 2, 3). We captured birds using monofilament leg nooses, mistnets and net launchers. Upon capture, we placed birds in individual cloth bags and fitted them with Argos (*n* = 55) or Argos-GPS (*n* = 86) transmitters that were either solar (*n* = 138) or battery powered (*n* = 3). (Microwave Telemetry, Inc., Columbia, MD, USA). We secured solar powered transmitters to birds with a teflon ribbon harness (Bally Ribbon Mills, Bally, PA) and glued external, battery powered transmitters to plastic neck collars (2 on bar-headed geese in Keoladeo National Park, India and 1 on a bar-headed goose in Chitwan National Park, Nepal). Transmitters ranged from 9.5 g to 70 g (Table 1) and average (± SE) weights were 2.1 ± 0.1% of the bird’s body mass prior to marking. After processing, we released birds near capture locations as soon as possible, usually within 1–4 hrs. We used capture, handling, and marking procedures approved by the USGS Patuxent Wildlife Research Center Animal Care and Use Committee.

**Table 1:** **Satellite telemetry data breakdown by species, marking location and date**

						**Transmitter**	
**Flyway**	**Species**	**Country**	**Marking site**	**Marking dates**	**Data type**	**Weights (g)**	***n***
Central Asian	BHGO	Mongolia	Terkhiin Tsagaan Lake	Jul ’08, Jul ’09	GPS	30	11
		China	Qinghai Lake	Mar ’07, Mar ’08	GPS	45	16
		Nepal	Chitwan National Park	Feb ’05	Argos*, GPS	30, 70	2^a^
		India	Pong Dam	Mar ’11	GPS	30	2^a^
		India	Keoladeo National Park	Feb ’05	Argos*, GPS	30, 70	5^a^
		India	Chilika Lake	Dec ’08	GPS	30	6
		India	Koonthankulam	Dec ’08, Jan – Feb ’09	GPS	30	5
	EUTE	India	Chilika Lake	Dec ’08	Argos	9.5	1
	EUWI	India	West Bengal	Dec ’09	Argos	12	3
	GADW	India	West Bengal	Dec ’09	Argos	12	2
	GARG	Bangladesh	Hakaluki Haor	Mar ’10, Mar ’11	Argos	9.5	4^b^
		India	West Bengal	Dec ’09 – Feb ’10	Argos	9.5	5^b^
		India	Chilika Lake	Dec ’08	Argos	9.5	2
		India	Koonthankulam	Dec ’08	Argos	9.5	4
	NOPI	Bangladesh	Hakaluki Haor	Mar ’11	Argos	9.5	3^c^
		India	West Bengal	Jan ’10	Argos	9.5	1^c^
		India	Koonthankulam	Dec ’08	Argos	12	2
	NOSH	Bangladesh	Hakaluki Haor	Mar ’10, Mar ’11	Argos	9.5, 12	5
		India	Chilika Lake	Dec ’08	Argos	12, 18	2
	RUSH	China	Qinghai Lake	Sep ’07, Mar ’08, Sep ’08	GPS	30, 45	23
		Bangladesh	Hakaluki Haor	Mar ’10	GPS	22	5^d^
		India	Brahmaputra River	Dec ’09	GPS	22	2^d^
		India	Chilika Lake	Dec ’08	GPS	30	1^d^
East Asian	EUTE	China	Poyang Lake	Mar ’07	Argos	12	3
	EUWI	China	Mai Po	Dec ’08, Dec ’09	Argos, GPS	12, 22	5
	FATE	China	Poyang Lake	Mar ’07	Argos	12, 18	4
	GARG	China	Poyang Lake	Mar ’07	Argos	12	1
	NOPI	China	Mai Po	Dec ’08, Dec ’09	Argos, GPS	12, 18, 22	10
	SWGO	Mongolia	Delger Tsagaan Lake	Aug ’06, Jul ’08	GPS	30, 70	4
	WHSW	Mongolia	Delger Tsagaan Lake	Aug ’06	GPS	70	2

Sample size refers to the number of birds included in the analyses. Species include bar-headed goose (BHGO), Eurasian teal (EUTE), Eurasian wigeon (EUWI), falcated teal (FATE), gadwall (GADW), garganey (GARG), northern shoveler (NOSH), northern pintail (NOPI), ruddy shelducks (RUSH), swan goose (SWGO), whistling swan (WHSW). ^a^Individual BHGO UDs from these three sites were grouped together in the single-species flyway. ^b^Individual GARG UDs from these two sites were grouped together in the multi-species flyway. ^c^Individual NOPI UDs from these two sites were grouped together in the multi-species flyway. ^d^Individual RUSH UDs from these three sites were grouped together in the population-level and multi-species flyways. *Battery powered, collar-mounted PTTs.

### Assigning locations to annual cycle stage

We used the complete sequence of locations that occurred between the breeding and wintering areas to estimate UDs for spring and fall migrations, only including migration events that spanned the entire distance between the two areas. Because we marked 11 different Anatidae species across a wide range of latitudes, there was considerable variation in migration phenology. We assigned locations to annual cycle stages (wintering, spring migration, breeding, fall migration) based on geographic area, scale of movement, arrival and departure dates, and comparisons of these metrics to those of conspecifics from the same marking site. Within and across marked species in our study, individual birds differed in their migration strategies. Consistent with many waterfowl species, some birds molted in close proximity to their breeding area, while others traveled hundreds of kilometers to separate wetlands [36].

### Data filtering and preparation

We received telemetry data from the Argos satellite tracking system (CLS America Inc., Largo, MD, USA). The average time between consecutive Argos locations was 22.4 ± 1.7 hr, while the average time between consecutive GPS locations was 4.5 ± 1.9 hr. To improve accuracy of Argos data, we used the Douglas Argos-Filter Algorithm ‘hybrid’ filter designed to account for sedentary periods (staging) interspersed with rapid, directional movement (migration) [37]. We retained only the highest quality location in each hour for Argos satellite data, allowing us to truncate the timestamp at the nearest hour and minimize computation time of dBBMMs. We used published error estimates for Argos locations of free-ranging waterfowl (based on concurrent [<5 min] GPS locations; [37]) to calculate mean error values for each Argos location class (3, 2, 1, 0, A, B, Z) for data filtered by the Douglas Argos-Filter Algorithm ‘hybrid’ filter. These mean error values ranged from 0.45 km (location class 3) to 7.92 km (location class B) and served as dBBMM parameter inputs. We used a location error of 23.5 m for GPS data [38].

### Use of dBBMMs

We ran dBBMMs using the ‘move’ package [39] in Program R [40] to estimate one UD for each full migration event in our data. In a sequence of three locations, the dBBMM assumes constant movement between the first and third location, which are connected by a Brownian bridge, while the second location is treated as an independent observation. The dBBMM estimates σ^2^_m_ for these three locations by maximizing the likelihood of observing the second location assuming random movement between successive locations and normally distributed location errors. The dBBMM identifies changes in movement speed and direction along the entire movement path and for user-defined subsets (windows) of locations, it calculates separate σ^2^_m_ values that correspond to these different movement behaviors. Within a sliding window with *w* locations, the dBBMM determines whether there is a behavioral change by comparing model fit using one or two estimates of σ^2^_m_. Specifically, the model uses Bayesian Information Criterion values to compare the log-likelihood of using one σ^2^_m_ value for the whole window with the log-likelihood of a window split into two parts at a breakpoint located anywhere within the window. Because σ^2^_m_ estimation requires at least three locations, the dBBMM requires a margin (*m*) with a minimum of three locations at the start and end of each window in which no breakpoints can be estimated. Larger window sizes increase reliability in σ^2^_m_ estimation but also increase the chance of missing short term changes in behavior. Larger margin sizes enhance the power to identify behavioral changes in the sliding window but increase the chance of missing breakpoints in the margin [29].

We calculated all UD output grids for GPS and Argos birds at the same spatial extent and at a 10 km^2^ grid resolution. We used a window size of 31 locations and margins of 11 locations for both GPS and Argos satellite data in all analyses based on Kranstauber et al. [29] and visual inspection of example results from our own data. This corresponded to a window length of approximately six days for GPS data and 28 days for Argos data.

### Creating individual-level routes from multiple migration events

For birds with multiple full migration events recorded (*n* = 41), we summed UDs from individual migration events to create migration routes that estimated relative use at the individual level throughout the entire year. The duration of migration events varied widely (range: <24 hr–184 d) by individual, species, and marking site. To account for this variation when summing multiple UDs, we weighted each individual UD by its migration event duration, multiplying all pixel values in a UD by the total number of days elapsed during its associated migration event. We summed the pixel values of all their weighted UDs, and then re-scaled their cumulative pixel values to sum to 1. The resulting UD represented the proportional amount of time occupied for each pixel across that bird’s entire migration route based on movement data from all available seasons [28]. For birds that molted in a site separate from their breeding area, we included locations representing post-breeding movements as part of the fall migration event but excluded locations from molting period itself.

### Creating routes at the population, species and multi-species levels

We used this same time-weighting and re-scaling procedure to produce population-level migration routes from individual routes, weighting individual bird UDs by migration event duration, summing these weighted UDs and re-scaling the cumulative pixel values for the resulting population-level UD. We grouped individuals into populations based on geographic proximity of marking sites (Table 1). Population-level UDs represent an estimate of the relative use during migration for each pixel across all marked birds in the population. We weighted each population-level UD by its average migration event duration, summed these weighted population UDs and re-scaled the resulting surface to estimate a single-species flyway route. Finally, we time-weighted and summed single-species routes to create multi-species migration routes for the CAF and EAAF. Because birds with multiple full migration events recorded showed fidelity to migration corridors across seasons and years, we did not calculate UDs for separate seasons at the population, species or multi-species level.

We calculated cumulative probability contours for each UD at the population, species and multi-species level to display migration routes on a map. We assumed UD values within the 50% contour were stopover sites used for resting and feeding over multiple days, those between the 50% and 75% contours were core movement areas characterized by short flights and frequent stops, and those between the 75% and 99% contours were flight corridors with minimal stops.

### Sensitivity analysis

We ran a sensitivity analysis in Program R that estimated the degree to which our samples of marked birds were representative of real-world flyways. For each subsample of *k* marked birds chosen from the total sample size of *n* marked birds (starting at *k* = 1 and continuing to *k = n* in increments of 3), we calculated the average %VI between (1) each of the volumes of multi-individual UDs constructed from a random sample (up to 100) of all possible combinations of *k* individuals, and (2) the volume of the overall flyway UD constructed from *n* marked birds in a flyway. We then fitted a smoothed curve to the data using a locally weighted regression (LOESS; [41]). If the final aggregated UDs depicted in Figures 1, 2 and 3 were accurate representations of real-world flyways, the curves would asymptote near 100% VI before reaching 100% of *n*.

## Additional file

Additional file 1: Figure S1. Percent volume of intersection between subsamples of aggregated individual UDs and overall flyway UDs. Individual curves correspond to two population-level ruddy shelduck routes, one species-level bar-headed goose route, and two multi-species routes (Central Asian and East Asian-Australasian Flyways).
