# Supplementary figures and images for: Mapping migratory flyways in Asia using dynamic Brownian bridge movement models
Source: Mov Ecol. 2015 Feb 2;3(1):3. doi: 10.1186/s40462-015-0029-6 (PMC4337761; doi:10.1186/s40462-015-0029-6)

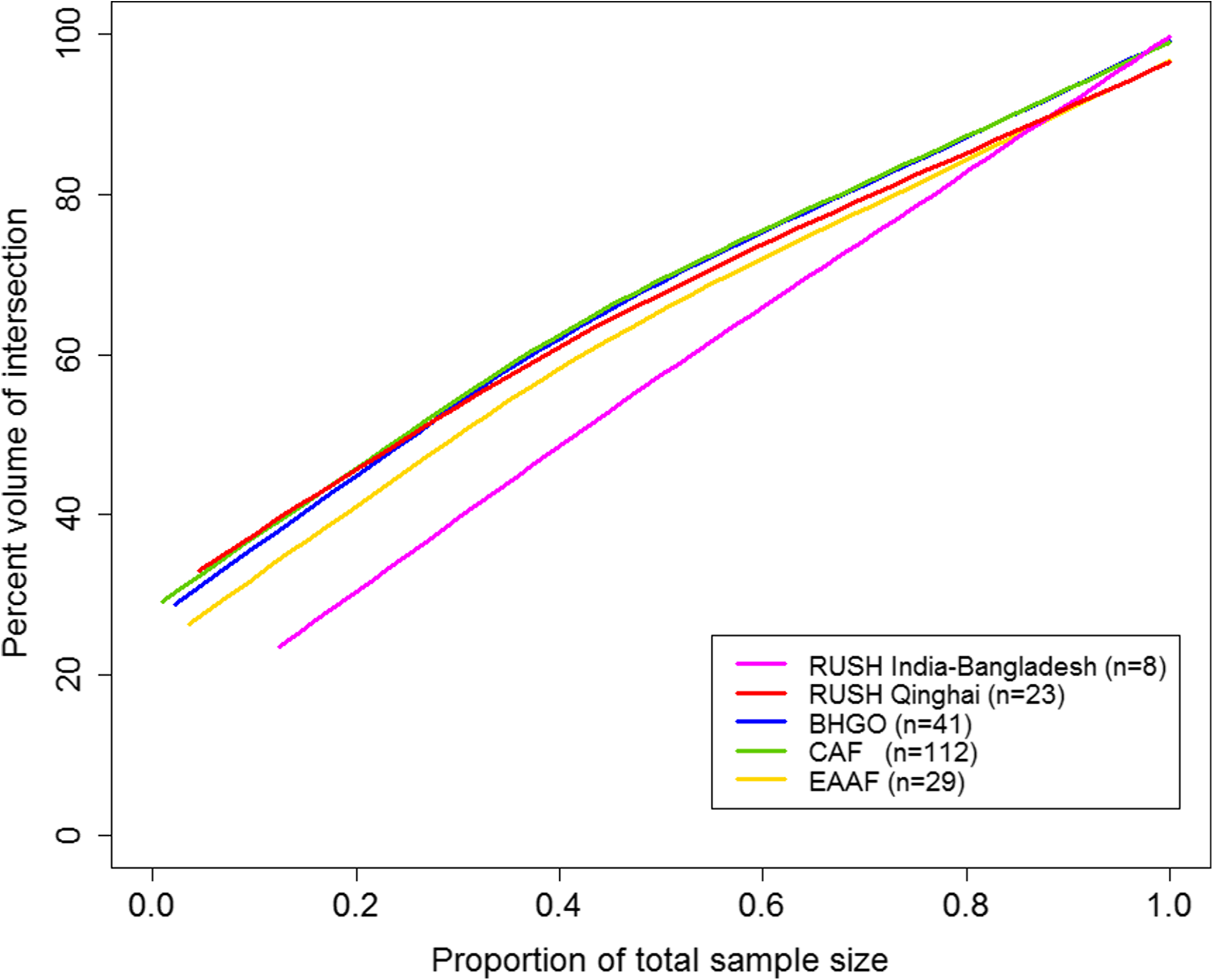

Supplement: Additional file 1: Figure S1. — Percent volume of intersection between subsamples of aggregated individual UDs and overall flyway UDs. Individual curves correspond to two population-level ruddy shelduck routes, one species-level bar-headed goose route, and two multi-species routes (Central Asian and East Asian-Australasian Flyways). [file 40462_2015_29_MOESM1_ESM.tif]
